# Moroccan *Leishmania infantum*: Genetic Diversity and Population Structure as Revealed by Multi-Locus Microsatellite Typing

**DOI:** 10.1371/journal.pone.0077778

**Published:** 2013-10-17

**Authors:** Ahmad Amro, Salsabil Hamdi, Meryem Lemrani, Idrissi Mouna, Hida Mohammed, Sabri Mostafa, Mohamed Rhajaoui, Omar Hamarsheh, Gabriele Schönian

**Affiliations:** 1 Faculty of Pharmacy, Alquds University, Jerusalem, Palestine; 2 Institut Pasteur du Maroc, Laboratoire des Leishmanioses, Casablanca, Morocco; 3 Faculty of Medicine and Pharmacy, University Sidi Mohammed ben Abdellah Fes, Morocco; 4 Laboratory of Parasitology, Ibn Sina Hospital, Rabat, Morocco; 5 Parasitology Department, Institut National d’Hygiène, Rabat, Morocco; 6 Department of Biological Sciences, Al-Quds University, Jerusalem, Palestine; 7 Institute of Microbiology and Hygiene, Charité University Medicine, Berlin, Germany; Federal Institute for Vaccines and Biomedicines, Germany

## Abstract

*Leishmania infantum* causes Visceral and cutaneous leishmaniasis in northern Morocco. It predominantly affects children under 5 years with incidence of 150 cases/year. Genetic variability and population structure have been investigated for 33 strains isolated from infected dogs and humans in Morocco. A multilocus microsatellite typing (MLMT) approach was used in which a MLMtype based on size variation in 14 independent microsatellite markers was compiled for each strain. MLMT profiles of 10 Tunisian, 10 Algerian and 21 European strains which belonged to zymodeme MON-1 and non-MON-1 according to multilocus enzyme electrophoresis (MLEE) were included for comparison. A Bayesian model-based approach and phylogenetic analysis inferred two *L.infantum* sub-populations; **Sub-population A** consists of 13 Moroccan strains grouped with all European strains of MON-1 type; and **sub-population B** consists of 15 Moroccan strains grouped with the Tunisian and Algerian MON-1 strains. Theses sub-populations were significantly different from each other and from the Tunisian, Algerian and European non MON-1 strains which constructed one separate population. The presence of these two sub-populations co-existing in Moroccan endemics suggests multiple introduction of *L. infantum* from/to Morocco; (1) Introduction from/to the neighboring North African countries, (2) Introduction from/to the Europe. These scenarios are supported by the presence of sub-population B and sub-population A respectively. Gene flow was noticed between sub-populations A and B. Five strains showed mixed A/B genotypes indicating possible recombination between the two populations. MLMT has proven to be a powerful tool for eco-epidemiological and population genetic investigations of *Leishmania*.

## Introduction

Leishmaniasis constitutes a group of diseases caused by obligatory, intracellular, protozoan parasites of the genus *Leishmania*, that cause a spectrum of diseases, ranging from self-limiting, self-curing cutaneous leishmaniasis (CL) to visceral leishmaniasis (VL) with fatal spontaneous evolution [1]. In areas bordering the Mediterranean Sea, *Leishmania infantum* is the etiologic agent of VL, but can also cause CL. VL is endemic in these countries, affecting the most vulnerable young children and adults, normally associated with HIV/AIDS [2]. The recent global VL and CL incidence were estimated by *Alvar et al* in 2012. Based on these estimates, approximately 0.2 to 0.4 million VL cases and 0.7 to 1.2 million CL cases occur each year. More than 90% of global VL cases occur in just six countries: India, Bangladesh, Sudan, South Sudan, Brazil and Ethiopia [2].Most strains of *L. infantum* isolated from Mediterranean foci belong to the predominant zymodeme MON-1 [3], despite their very wide geographical distribution. However, other *L. infantum* zymodemes have been identified in this region: MON-11, MON-24, MON-27, MON-33, MON-34, MON-37, MON-72, MON-77, MON-80, MON-98, MON-105, MON-108 and MON-199. Epidemiological patterns of leishmaniasis in Mediterranean area are changing dramatically, due to several factors, such as widespread migration from rural to urban and peri-urban areas, socio-political situation [4,5], climatic changes increasing exposure to the sandfly and also, in urban areas, increasing HIV infection.

**Figure 1:**
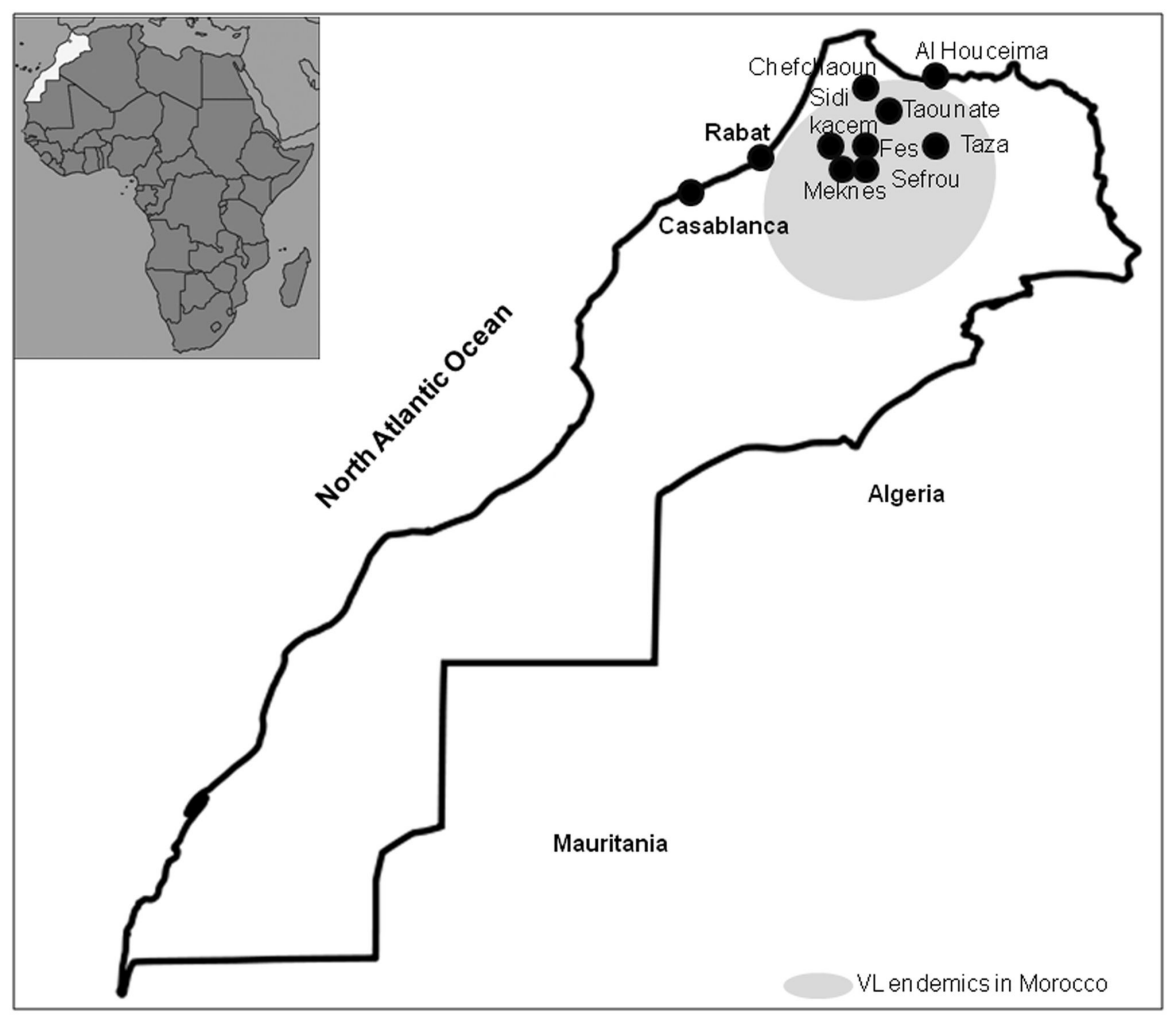
Map of Morocco with all areas endemic for VL (shadowed areas).

VL is widespread in northern Morocco (Chefchaoun, Taounate, Taza, Fes, My yacoub, Meknes, Sefrou, Al Houceima and sidi kacem) (Figure 1). Few epidemiological data are available concerning its epidemiology and clinical features. Also a new focus of CL due to *L. Infantum* was recently described [6]. Before 1995, human VL was not an obligatory reportable disease. The incidence of VL was over 150 cases/years in 2006-2008 [2] of which children under 4 years old are most affected. Expansion of arid zone and increase of global temperature increases the incidence of leishmaniasis in this region [7]. Dog is the main reservoir of *L.infantum* in Morocco as in all the Mediterranean basin [8,9]. The sand fly vectors of *L. infantum* in this region are: *P. perniciosus* [10], *P*.*longicuspis* [11] and *P. ariasi* [9]. Zymodeme MON-1 is predominant in Morocco, however, zymodeme MON-24 has been occasionally isolated from a dog [12]. This zymodeme was considered to cause CL sporadically.

Different methods have been used for the identification and classification of *Leishmania* parasites as reviewed [5]. These methods, including multilocus enzyme electrophoresis (MLEE) which is still considered as the gold standard for species and strain typing of *Leishmania*, are limited in the intrinsic level of polymorphism they can detect and are, only in exceptional cases, able to differentiate strains in the zymodeme MON-1 [5]. Epidemiological studies on VL caused by *L. infantum* require the use of highly discriminative techniques that are able to differentiate MON-1 strains. Multilocus Microsatellite Typing (MLMT) has proven to be a powerful tool for population genetic and epidemiological studies of *Leishmania* *spp*. For review see Schoenian et al.[5]. Ochsenreither et al. [13], Kuhls et al. [14,15] developed panel of 14 microsatellite markers which are able to discriminate *L. Infantum* strains and have been useful for investigating genetic polymorphism and population structure of *L.infantum* in various endemic areas [16-18].

In the present study, we used the above mentioned 14 microsatellites markers to investigate the genetic polymorphism and population structure of Moroccan *L. infantum* strains isolated from human and canine cases (CanL). We have compared the Moroccan MLMT profiles to those of Tunisian, Algerian and European strains belonging to previously characterized MON-1 and non-MON-1 isoenzyme groups. This comparison was done to understand VL transmissions scenarios in North Africa and South Europe.

## Material and Methods

### 1: Source of *Leishmania* and DNA extraction

Thirty-three strains of Moroccan *L. infantum* were analysed. Thirty strains were isolated from human VL cases aged between 1-7 years old, and three strains from canine cases. The strains came from all endemic areas in Northern Morocco; 11 from Taounate, eight from Fes, seven from My Yacoub, one from Al Houceima, one from Taza, and for five strains the exact origin was unknown. Table 1 list the strains, their origin and geographical distribution, the clinical form of the disease, and the source of strains used in this study and the reference strains. Isoenzyme profiles were available for only 2 Moroccan strains which belonged to the MON-1 zymodeme.

**Table 1:** *L.infantum* strains that have been analysed in this study.

**WHO-Code**	**Country**	**Origin**	**Zymodeme**	**clinical form**	**Age**	**Structure**	**Source**
MRO 1	Morocco	Fes	**nd**	VL	2 years	Sub-pop A	This study
MRO 7	Morocco	Fes	**nd**	VL	3 years	Sub-pop A	This study
MRO 8	Morocco	Taounate	**nd**	VL	**nd**	A/B mix	This study
MRO 10	Morocco	**ND**	**nd**	VL	**nd**	Sub-pop A	This study
MRO 13	Morocco	**ND**	**nd**	CanL	**nd**	Sub-pop A	This study
MRO 15	Morocco	**ND**	**nd**	CanL	**nd**	Sub-pop A	This study
MHOM/MA/2004/BOU	Morocco	My Yacoub	**nd**	VL	nd	Sub-pop B	This study
MHOM/MA/2004/CHOUI	Morocco	Taounate	**nd**	VL	2 years	Sub-pop A	This study
MHOM/MA/2003/NAB	Morocco	Taounate	**nd**	VL	1 years	Sub-pop B	This study
MHOM/MA/2004/SER	Morocco	My Yacoub	**nd**	VL	2 years	A/B mix	This study
MHOM/MA/2000/KHAL	Morocco	Fes	**MON-1**	VL	nd	Sub-pop B	This study
MHOM/MA/2003/LAKH	Morocco	Taounate	**nd**	VL	2 years	Sub-pop B	This study
MHOM/MA/2003/ZAHI	Morocco	Taounate	**nd**	VL	3 years	Sub-pop B	This study
MHOM/MA/2003/NAW	Morocco	Taounate	**nd**	VL	3 years	Sub-pop B	This study
MHOM/MA/2004/NAY	Morocco	My Yacoub	**nd**	VL	1 years	Sub-pop A	This study
MHOM/MA/2003/CHOU	Morocco	Taounate	**nd**	VL	2 years	Sub-pop B	This study
MHOM/MA/2003/BER	Morocco	Fes	**nd**	VL	2 years	Sub-pop B	This study
MHOM/MA/2003/ELO	Morocco	Taounate	**nd**	VL	2 years	Sub-pop B	This study
MHOM/MA/2003/ANS	Morocco	Fes	**nd**	VL	1 years	Sub-pop A	This study
MCAN/MA/1996/77	Morocco	My Yacoub	**MON-1**	CanL	4 years	A/B mix	This study
MHOM/MA/2005/HAJ	Morocco	Taounate	**nd**	VL	2 years	Sub-pop B	This study
MHOM/MA/2005/CHA	Morocco	My Yacoub	**nd**	VL	2 years	Sub-pop B	This study
MHOM/MA/2005/ZAG	Morocco	My Yacoub	**nd**	VL	3 years	Sub-pop A	This study
MHOM/MA/2005/ZEM	Morocco	Taounate	**nd**	VL	3 years	A/B mix	This study
MHOM/MA/2006/RZI	Morocco	Fes	**nd**	VL	7 years	Sub-pop B	This study
MHOM/MA/2006/NAB	Morocco	Fes	**nd**	VL	2 years	Sub-pop B	This study
MHOM/MA/2003/BOUZ	Morocco	ND	**nd**	VL	4 years	Sub-pop A	This study
MHOM/MA/2003/TAB	Morocco	Taza	**nd**	VL	3 years	Sub-pop A	This study
MHOM/MA/2003/BOUS	Morocco	ND	**nd**	VL	2 years	Sub-pop A	This study
MHOM/MA/2004/FAT	Morocco	Fes	**nd**	VL	3 years	Sub-pop B	This study
MHOM/MA/2005/MAR	Morocco	Al Houceima	**nd**	VL	1 years	A/B mix	This study
MHOM/MA/2003/BEN	Morocco	Taounate	**nd**	VL	1 years	Sub-pop B	This study
MHOM/MA/2005/HMI	Morocco	My Yacoub	**nd**	VL	5 years	Sub-pop A	This study
MCAN/FR/1987/RM1	France	Marseille	108	CanL	**nd**	Sub-pop A	Control Strain
MHOM/FR/1978/LEM75	France	Languedoc	1	VL	**nd**	Sub-pop A	Control Strain
MHOM/FR/1995/LPN114	France	Cote d'Azur	1	VL	**nd**	Sub-pop A	Control Strain
MHOM/FR/1997/LSL29	France	Languedoc	1	CL	**nd**	Sub-pop A	Control Strain
MHOM/GR/2001/GH1	Greece	Athens	1	VL	**nd**	Sub-pop A	Control Strain
MHOM/GR/2001/GH2	Greece	Athens	1	VL	**nd**	Sub-pop A	Control Strain
MHOM/GR/2001/GH5	Greece	Crete	1	VL	**nd**	Sub-pop A	Control Strain
MHOM/GR/2001/GH6	Greece	Athens	98	VL	**nd**	Sub-pop A	Control Strain
MHOM/PT/2000/IMT260	Portugal	Lisbon	1	CL	**nd**	Sub-pop A	Control Strain
MCAN/ES/1986/LEM935	Spain	Poboleda	77	CanL	**nd**	Sub-pop A	Control Strain
MHOM/ES/1993/PM1	Spain	Mallorca	1	VL/HIV+	**nd**	Sub-pop A	Control Strain
MHOM/ES/1986/BCN16	Spain	Catalonia	1	CL	**nd**	Sub-pop A	Control Strain
MHOM/FR/1962/LRC-L47	France	NA	NA	VL	**nd**	Population 2	Control Strain
MHOM/FR/1996/LEM3249	France	Roussillion	29	CL	**nd**	Population 2	Control Strain
MHOM/FR/1980/LEM189	France	Roussillion	11	CL	**nd**	Population 2	Control Strain
MHOM/IT/1994/ISS1036	Italy	NA	228	VL	**nd**	Population 2	Control Strain
MHOM/ES/1987/Lombardi	Spain	NA	24	CL	**nd**	Population 2	Control Strain
MHOM/ES/1988/LLM175	Spain	Madrid	198	VL/HIV+	**nd**	Population 2	Control Strain
MHOM/ES/1991/LEM2298	Spain	Valencia	183	VL/HIV+	**nd**	Population 2	Control Strain
MHOM/ES/1992/LLM373	Spain	Madrid	199	VL/HIV+	**nd**	Population 2	Control Strain
MHOM/DZ/1999/LIPA979	Algeria	Tizi Ouzou	MON-1	VL	**nd**	Sub-pop B	Control Strain
MHOM/DZ/1999/LIPA1002	Algeria	Tizi Ouzou	MON-1	VL	**nd**	Sub-pop B	Control Strain
MCAN /DZ/2000/LIPA1109	Algeria	Alger	MON-1	CanL	**nd**	Sub-pop B	Control Strain
MCAN /DZ/2000/LIPA1113	Algeria	Alger	MON-1	CanL	**nd**	Sub-pop B	Control Strain
MCAN /DZ/2000/LIPA1117	Algeria	Alger	MON-1	CanL	**nd**	Sub-pop B	Control Strain
MHOM/DZ/1996/LIPA477	Algeria	Ain Defla	MON-24	CL	**nd**	Population 2	Control Strain
MHOM/DZ/1999/LIPA1058	Algeria	Lakhdaria	MON-80	CL	**nd**	Population 2	Control Strain
MHOM/DZ/2001/LIPA1140	Algeria	Boumerdes	MON-24	CL	**nd**	Population 2	Control Strain
MHOM/DZ/2001/LIPA1226	Algeria	Alger	MON-24	CL	**nd**	Population 2	Control Strain
MHOM/DZ/1995/LIPA459	Algeria	Lakhdaria	MON-24	CL	**nd**	Population 2	Control Strain
MHOM/TN/2001/Tus167	Tunisia	Monastir	1	VL	4 years	Sub-pop B	Control Strain
MHOM/TN/2002/20S	Tunisia	Beja	1	VL	9 months	Sub-pop B	Control Strain
MHOM/TN/2002/Tus221	Tunisia	Monastir	1	VL	3 years	Sub-pop B	Control Strain
MHOM/TN/2002/Tum222	Tunisia	Monastir	1	VL	3 years	Sub-pop B	Control Strain
MCAN/TN/2002/LCnJ20S	Tunisia	Tunis	1	CanL	2 years	Sub-pop B	Control Strain
MHOM/TN/2004/LC64	Tunisia	Tunis	24	CL	6 years	Population 2	Control Strain
MHOM/TN/2002/LC95	Tunisia	Béja	24	CL	15 years	Population 2	Control Strain
MHOM/TN/2002/SFC89	Tunisia	Sfax	24	CL	54 years	Population 2	Control Strain
MHOM/TN/2005/SFC51	Tunisia	Sfax	24	CL	50 years	Population 2	Control Strain
MHOM/TN/2004/TLC3	Tunisia	Siliana	24	CL	10 years	Population 2	Control Strain

VL, visceral leishmaniasis; CL, cutaneous leishmaniasis; CanL, canine leishmaniasis. Sub-pop A, Sub-pop B, mixed A/B and Population 1 represent populations according to STRUCTURE. WHO, World Health Organization

 For 27 strains, DNA was extracted from promastigotes grown in Novye MacNealeNicolle biphasic culture medium (NNN) [19]. Furthermore, DNA was isolated from amastigotes in bone marrow aspirates spotted on six glass slides. DNA extraction from cultures was done by using phenol—chloroform extraction method and from slides, with some modifications, as described previously [20,21]. Figure 1 shows geographical distribution of the studies strains and VL endemic areas in Morocco. 

The microsatellite profiles obtained previously for ten strains from Tunisia, ten from Algeria and 20 from different European countries were used as controls for comparison [15-17] and to investigate gene flow amongst North African and South European strains. These strains represent different clinical forms (CL and VL) and belonged to zymodeme MON-1 and different non- MON-1 zymodemes, see Table 1.

### 2: *L. infantum* identification and microsatellite genotyping

The internal transcribed spacer 1 (ITS-1) was amplified for all samples. The PCR product was digested with the restriction endonuclease HaeIII as described previously [22]. The resulting RFLP profile was identical to those of the WHO reference strain of *L. infantum* MHOM/TN/1980/IPT1 (data not shown). 

Microsatellite genotyping was done using the dinucleotide microsatellite markers: Lm2TG, TubCA, Lm4TA, Li41-56, Li46-67, Li22-35, Li23-41, Li45-24, Li71-33, Li71-5/2, Li71-7, LIST7031, LIST7039 and CS20. PCR conditions were described previously [13,14]. To expose microsatellite profiles, the microsatellite-containing fragments were analyzed by capillary electrophoresis (SMB Services in Molecular Biology Berlin) with an automated ABI PRISM GeneMapper sequencer (Applied Biosystems).

### 3: Microsatellite data analysis

Two models were used to analyse the microsatellite data: (1) Population structure analysis was investigated using the program STRUCTURE 2.2, which implements a Bayesian model-based clustering method, using genotype data consisting of unlinked markers [23]. The admixture model was used. Markov chain Monte Carlo (MCMC) searches consisted of a burn-in length of 10,000 iterations followed by a run of 100,000 replications for each setting of K (the number of populations) from 1 to 10 with 10 replicate runs of each. The most appropriate number of populations was determined based upon *ad hoc* statistic *⎢K* that evaluates the second order rate of change of the likelihood function with respect to the number of populations (K) [24]. (2) Microsatellite-based genetic distances analysis was based on the proportion of shared alleles distances. Phylogenetic trees were constructed using Neighbour-joining (NJ) method by the help of the softwares MSA 3.0, POPULATIONS 1.2.28 and MEGA version 3.1, as described previously [16].

Descriptive statistics for the observed genetic populations were calculated with the help of GDA software [24]. This includes allelic diversity (number of allelic variants per marker and mean number of alleles (MNA) per population), proportion of polymorphic loci (P), expected (He) and observed (Ho) heterozygosity and inbreeding coefficient (*F*_IS_). The degree of genetic differentiation and gene flow among populations were assessed by calculating *F*_*ST*_ values with corresponding *p*-values, FST values higher than 0.25 indicate strong genetic differentiation [25].

### 4: Ethics statement

According to ethical approval of this study, all samples were anonymized. Written informed consent was obtained from each study participant or the parents/guardians on behalf of the children under 15 years old. Study design and procedures were revised and approved by the Institutional Review Board (IRB), Institut Pasteur du Maroc, Casablanca, Morocco, and the Ethical Committee of Charitè University Medicine, Berlin, Germany. 

## Results

Every analysed Moroccan strain had an individual microsatellite profile (33 genotypes). Thirteen markers were polymorphic, only marker Li 71-33 was monomorphic. Marker Lm4TA was the most polymorphic one presenting seven alleles, whereas markers Li41-56, Li46-67, Li 71-5/2 and LIST7039 were least polymorphic presenting only two alleles for each. For the 33 Moroccan strains, the mean number of allelic variants per locus (*A*) was 3.21. The observed heterozygosity (Ho) was between 0 and 0.15 and the expected heterozygosity (He), representing the probability that an individual will be heterozygous over the loci tested, ranged from 0 to 0.81 and was in most cases much higher than Ho. Inbreeding coefficient per locus (*F*_IS_) was positive for 11 markers and ranges from 0.93 to .037, for the other three markers (*F*_IS_) values was zero. This indicates a large number of homozygotes in the investigated strains (data not shown). 

**Figure 2:**
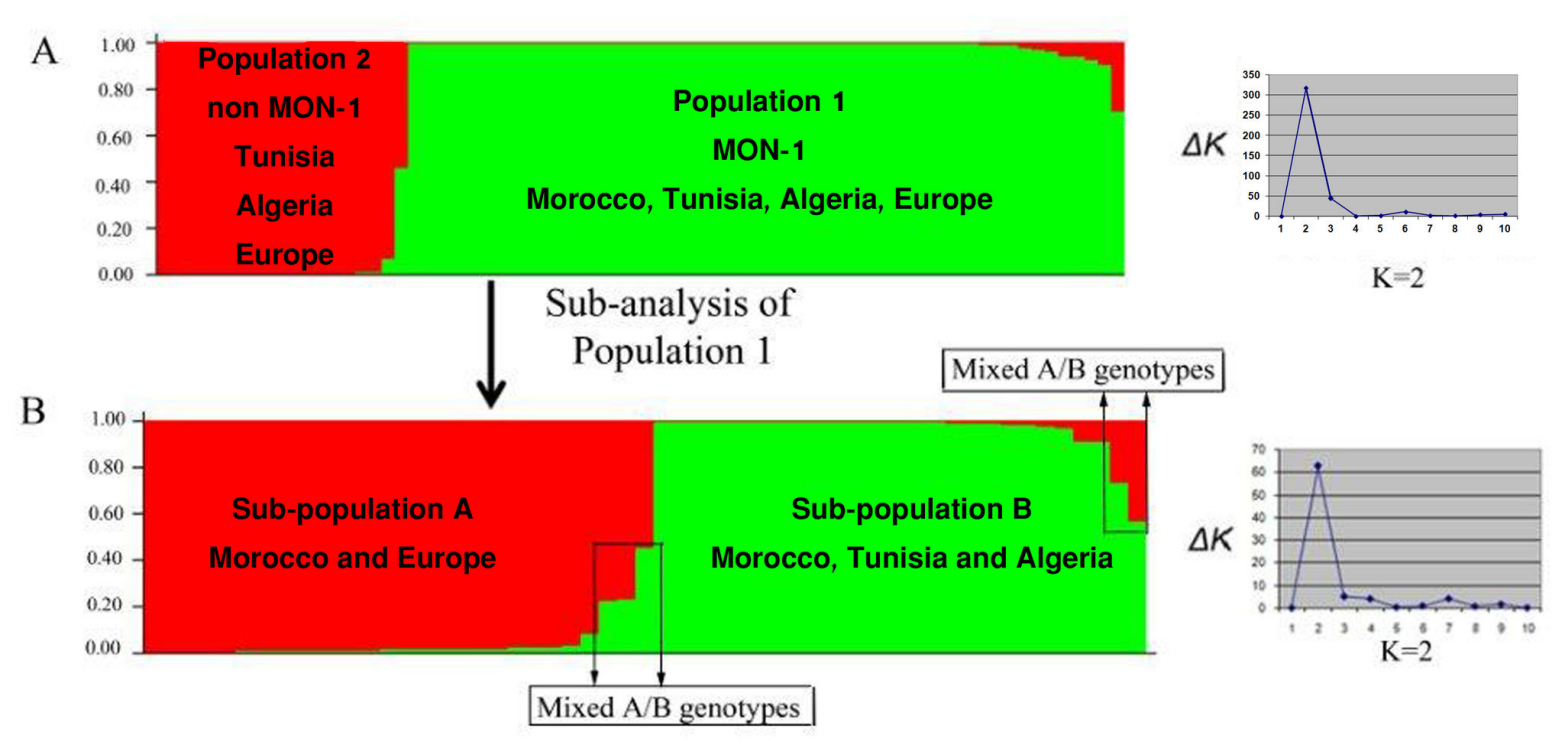
Estimated population structure for 33 Moroccan *L. infantum* strains as inferred by STRUCTURE software on the basis of data for 14 microsatellite markers. Each of the strains is represented by a single vertical line divided into K colors, where K is the number of populations assumed. Each color represents one population, and the length of the colors segment shows the strain’s estimated proportion of membership in that population.(A) the two main populations derived from the whole dataset which divided strains into MON-1 and non-MON-1 populations. (B) Sub-population analysis of **Population 1** (MON-1 group) shows two sub-populations. K represents the true number of populations and sub-populations.

Two main populations were detected by Structure for the data set analysed; **Population 1** consisted of all strains from Morocco and the Tunisian, Algerian and European strains of MON-1 type and those of MON-77, 89 and 108, closely related to MON-1 as described previously [15,18]. **Population 2** consisted of all non-MON-1 strains from Tunisia, Algeria and Europe which were added as controls; see Figure 2A and Table 1. These two populations were genetically different as shown by their *F*_ST_ value (0.35) and *P*-value 0.0001.

**Figure 3:**
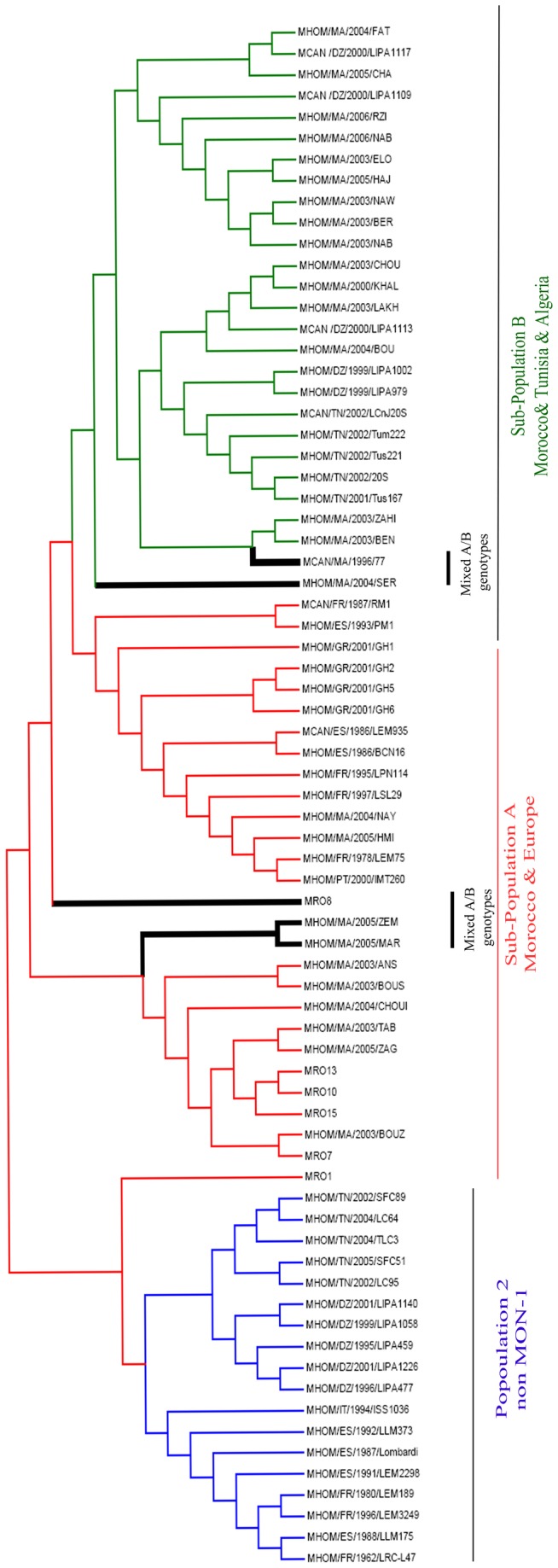
Unrooted neighbor-joining tree inferred from genetic distances derived from the proportions of alleles shared among 55 Moroccan *L. infantum* strains based on 14 microsatellites markers. Two main clusters were detected and the “MON-1” cluster was further sub-divided into two sub-clusters. This was in full agreement with the STRUCTURE results (Figure 2). Mixed 1A/1B genotypes were shown in intermediate positions in the tree.

When re-analysed separately by Structure, **Population 1** was subdivided into two sub-populations (Figure 2B). **Sub-population 1A** consisted of 13 Moroccan strains; three from Fes, three from My Yacoub, one from Taounate, one from Taza, five of unknown origin in the endemic area and all European strains of MON-1 type that have been added for comparison. **Sub-population 1B** comprised 15 Moroccan strains; eight from Taounate, five from Fes, two from My Yacoub, and all Tunisian and Algerian MON-1 control strains (Figure 2B and Table 1). These two sub-populations were as well significantly different as shown by their *F*_ST_ value (0.34) and *P*-value 0.0001. Five Moroccan strains have shown mixed genotypes between sub-populations 1A and 1B; two strains from My Yacoub, two from Taounate and one from Al Houceima (see Table 1, Figure 2 and Figure 3). Theses strains shared allele’s characteristic for each sub-population and showed significant shared membership in both sub-populations (Table 2). 

**Table 2:** Membership coefficients of mixed genotypes between **Sub-population 1A** and **1B** as calculated by the STRUCTURE program.

WHO code	Sub-population 1A	Sub-population 1B
MHOM/MAR/2004/SER	0.431	0.569
MCAN/MAR/1996/77	0.267	0.733
MHOM/MAR/2005/ZEM	0.738	0.262
MHOM/MAR/2005/MAR	0.759	0.241
MRO 8	0.532	0.468

Measures for genetic diversity have been calculated for the two Populations **1** and **2** and for **Sub-populations 1A** and **1B**, the five mixed genotypes were excluded from this analysis (Table 3). **Population 2** (non-MON-1) was found to be more diverse than **Population 1**, while **Sub-population 1A** was more diverse than **Sub- population 1B** (Table 3). Inbreeding coefficient (*F*_IS_) was highest for **Population 1** (MON-1 strains), and equally for both sub-populations of this population (Table 3). 

**Table 3:** Genetic diversity and characterization of the *L. infantum* populations and sub-populations found by STRUCTURE in this study.

Populations	n	p	MNA	He	Ho	*F*_IS_
Population 1 (MON-1)	55	0.85	4.64	0.39	0.06	0.84
Population 2 (non MON-1)	18	1	7.28	0.79	0.35	0.55
Sub-pop alnalysis with mixed strains being excluded						
Sub-pop A	25	0.92	3.85	0.414	0.07	0.82
Sub-pop B	25	0.5	2.28	0.2	0.03	0.83

n - number of strains; P - proportion of polymorphic loci; MNA - mean number of alleles; He - expected heterozygosity; Ho - observed heterozygosity; *F*_IS_ - inbreeding coefficient.

Three main clusters were detected in the NJ distance tree based on the proportion of shared alleles (*D*_AS_) measure (Figure 3). The first cluster comprised all non-MON-1 strains from Tunisia, Algeria and Europe and was identical with **Population 1**. The second cluster consisted of 13 Moroccan and all European MON-1 strains and was congruent with **Sub-population 1A**. Finally, the third cluster included the same 15 Moroccan strains and Tunisian and Algerian MON-1 strains as **Sub-population 1B** did. Interestingly, the mixed genotypes (five Moroccan strains) formed a separate cluster which took an intermediate position between and within the two sub-populations A and B. 

Strains isolated from canine cases grouped together with strains from humans in **Sub-population 1A**. No correlation was found between a particular MLMT profile and host background.

## Discussion

In this study, a panel of 14 microsatellite markers was applied to investigate the genetic polymorphism and population structure of Moroccan *L. infantum* strains and to compare them with strains from North African and European countries. 

Both phylogenetic analysis based on genetic distances as well as Structure analysis using a Bayesian clustering approach grouped the Moroccan strains together with MON-1 strains or strains closely related to MON-1 from Tunisia, Algeria and Europe in Population 1. None of the Moroccan strains were assigned to the non MON-1 population (**Population 2**). Isoenzyme analysis had been performed for only two of the Moroccan strains studied which were typed as MON-1. MLMT analysis suggests that the remaining Moroccan strains analysed here also represent MON-1 and/or MON-1 closely related zymodyme types. 

Surprisingly, two sub-populations were detected in Morocco. **Sub-population 1A** consisted of 13 Moroccan strains and that were related to the European MON-1 strains added for comparison. On the other hand, **Sub-population 1B** consisted of 15 Moroccan strains that were more similar to the Tunisian and Algerian MON-1 strains included in this study. The two sub-populations were genetically different from each other as confirmed by their *F*_ST_ value and the corresponding *P*-value. 

Phylogenetic analysis based on proportion of shared alleles was compatible with structure analysis based on Bayesian clustering model. The detected clusters were identical with identified populations and sub-populations hence confirming the presence of two sub-populations of *L. infantum* in Morocco and the presence of gene flow and mixed genotypes between them.

The presence of two *L. infantum* sub-populations co-existing in Moroccan endemic foci suggests multiple introduction of *L. infantum* from/to Morocco. Two scenarios are possible: (1) introduction from/to the neighboring North African countries, this is supported by the presence of **Sub-population 1B** (2), introduction from/to the European Mediterranean countries, this is supported by the presence of **Sub-population 1A**. Since Morocco is the African country closest to the European continent, through the Strait of Gibraltar, and due to the frequent migration of humans and their animals from and to Europe, back and forth introduction of the disease cannot be excluded. Due to their high variability and extensive size homoplasy, microsatellite markers are not suitable for estimating evolutionary history and are not able to confirm the origin and direction of introduction of Moroccan *L. infantum* from/to Europe. However, genomic sequencing data from North African and European strains is needed to verify these scenarios.

The presence of mixed genotypes between the Moroccan sub-populations indicates the presence of gene flow between them. These strains might represent either mixed infections or 1A/1B hybrid strains. Since all mixed Moroccan genotypes were isolated from amastigotes in bone marrow aspirate spotted on slides, cloning of these strains to test whether they are true hybrids was not possible. Hybrids seem to occur quite frequently. Previous studies in Algeria [16] and Tunisia [17] revealed hybrid genotypes for 12 of the 82 Algerian and Tunisian strains (14.6% ) with one Tunisian strain being cloned and proven as real hybrid. Since multiple heterozygous loci were identified among hybrids and gene flow was detected between different populations, recombination between strains with different alleles seems to be the most parsimonious explanation [26]. The occurrence of gene flow and genetic recombination is increasingly being suggested for different *Leishmania* species [27] although the mechanism of genetic exchange remains to be established. As *L. infantum* infects humans and dogs and continues its life cycle in sandfly midgut, it is vital to investigate the compartments where genetic recombination of *Leishmania* could take place. It was recently reported that *L. major* is capable of having asexual cycle consistent with a meiotic process in sandfly vector [28]. 

Three sandfly species transmit *L. infantum* in Morocco; *P. perniciosus* [10], *P*.*longicuspis* [11] and *P. ariasi* [9]. One can speculate whether the sub-populations of Moroccan *L. infantum* might be related to the existence of different sand fly vector species involved in transmitting the respective parasites, as previously described for *L .tropica*, in North Israel [29]. To prove this hypothesis, it is needed to isolate strains of *L. infantum* from the respective sandfly species and investigate them with MLMT. Moreover, a new focus of cutaneous leishmaniasis due to *L. Infantum* was recently described in Morocco. A clear relationship between the clinical presentation (VL vs CL) of leishmaniasis and parasite genotype could be demonstrated in Algeria [16] and Tunisia [17]. All Moroccan VL and CanL cases analyzed in this study have shown typical symptoms of visceral and canine leishmaniasis without significant differences in disease outcome between cases belonging to the two sub-populations, however, *L. infantum* strains that have caused CL in Morocco have not yet been analyzed by MLMT . Human and canine strains presented identical MLMT profiles. This highlights the key role of dogs as reservoirs of *L.infantum* in Morocco.

To our knowledge, this is the first study that investigates the population structure and genetic diversity of *L. infantum* in Morocco by applying MLMT. We were able to detect two different sub-populations of *L. infantum* in Morocco and correlated them with strains isolated from North African countries and Europe. Putative hybrid strains indicated the presence of gene flow among the two sub-populations. However, it is needed to investigate more strains representing different hosts, clinical forms and zymodyme types to better understand the overall population structure and molecular epidemiology of *L. infantum* in Morocco and the other North African countries.
